# DAY-TO-DAY VARIABILITY BETWEEN AFFECT AND LEISURE ACTIVITY ENGAGEMENT IN BLACK ADULTS

**DOI:** 10.1093/geroni/igac059.801

**Published:** 2022-12-20

**Authors:** Angie Sardina, Alyssa Gamaldo, Jason Allaire, Keith Whitfield

**Affiliations:** UNC Wilmington, Wilmington, North Carolina, United States; Pennsylvania State University, State College, Pennsylvania, United States; North Carolina State University, Raleigh, North Carolina, United States; University of Nevada, Las Vegas, Las Vegas, Nevada, United States

## Abstract

This study examined daily variability in affect and leisure engagement, within community-dwelling Black adults (age range=50-80 years). Associations between affect and leisure engagement, and moderating sociodemographic factors, were explored. Fifty adults (78% women; M education=11.62) reported affect and leisure engagement across 8-occasions over 2-3 weeks. Participants averaged 3-leisure activities/day with more engagement in watching TV, walking, reading, and visiting others. Significant within-person variation across daily affect and domains of leisure engagement were observed. Greater negative affect was significantly associated with lower social and total leisure engagement, particularly for adults with lower levels of education (p’s<.05). Results demonstrate within-person changes in the type of leisure engagement among Black adults, with potential factors related to interconnections between affect and sociodemographics (e.g., education). This study reveals promising directions for future research to implement models estimating both between- and within-person effects of daily leisure engagement, within minority populations and across racial groups.

